# Personalized trimodal prehabilitation for gastrectomy

**DOI:** 10.1097/MD.0000000000020687

**Published:** 2020-07-02

**Authors:** Augustinas Bausys, Martynas Luksta, Justas Kuliavas, Giedre Anglickiene, Vyte Maneikiene, Lina Gedvilaite, Jelena Celutkiene, Ieva Jamontaite, Alma Cirtautas, Svetlana Lenickiene, Dalia Vaitkeviciute, Edita Gaveliene, Gertruda Klimaviciute, Rimantas Bausys, Kestutis Strupas

**Affiliations:** a Clinic of Gastroenterology, Nephrourology and Surgery, Institute of Clinical Medicine, Faculty of Medicine, Vilnius University; b Department of Medical Oncology, National Cancer Institute; c Clinic of Cardiac and Vascular Diseases, Institute of Clinical Medicine of the Faculty of Medicine; d Department of Rehabilitation, Physical and Sports Medicine, Institute of Health Sciences, Faculty of Medicine, Vilnius University; e National Cancer Institute, Vilnius, Lithuania.

**Keywords:** gastric cancer, home-based, prehabilitation, randomized control trial

## Abstract

**Background:**

Surgery is the only potentially curative treatment for gastric cancer, however, it bears a high postoperative morbidity and mortality rate. A recent randomized control trial proposed prehabilitation to reduce the postoperative morbidity in patients undergoing major abdominal surgery. Currently, there is a lack of evidence of using prehabilitation for patients undergoing gastrectomy for gastric cancer. The aim of our study is to demonstrate that home-based prehabilitation can reduce postoperative morbidity after gastrectomy for gastric cancer.

**Methods:**

PREFOG is a multi-center, open-label randomized control trial comparing 90-days postoperative morbidity rate after gastrectomy for gastric cancer between patients with or without prehabilitation. One-hundred twenty-eight patients will be randomized into an intervention or control group. The intervention arm will receive trimodal home-based prehabilitation including nutritional, psychological and exercise interventions. Secondary outcomes of the study will include physical and nutritional status, anxiety and depression level, quality of life, postoperative mortality rates and full completion of the oncological treatment as determined by the multidisciplinary tumor board.

**Discussion:**

PREFOG study will show if home-based trimodal prehabilitation is effective to reduce postoperative morbidity after gastrectomy for gastric cancer. Moreover, this study will allow us to determine whether prehabilitation can improve physical fitness and activity levels, nutritional status and quality of life as well as reducing anxiety and depression levels after gastrectomy for gastric cancer.

**Trial registration:**

ClinicalTrials.gov NCT04223401 (First posted: 10 January 2020).

## 1 Introduction

Surgery is the main and only curative treatment option for gastric cancer (GC).^[1]^ Despite the progress in surgical and anesthetic techniques, gastrectomy remains associated with high postoperative morbidity (∼50%) and mortality (∼5%) rates.^[2–4]^ Furthermore, patients suffering postoperative complications are less likely to receive adjuvant therapy or must delay the initiation of it^[5,6]^ and it impairs the long-term outcomes.^[7]^ Therefore, there is a great need for novel strategies to reduce the postoperative morbidity after gastrectomy for GC.

Poor physical condition (determined by cardiopulmonary exercise testing), sarcopenia, and preoperative malnutrition often accompanies GC and represents decreased physiological reserve, predicting postoperative complications.^[4,8,9]^ Moreover, the majority of patients with resectable GC are considered for perioperative chemotherapy which improves oncological outcomes but impairs patients’ physical fitness before the surgery.^[10,11]^ Some patients’ risk factors are modifiable and may be improved within several weeks before surgery by a short multimodal prehabilitation consisting of physical training, nutritional adjustments and psychological support.^[12–14]^ Besides improved physical and nutritional status attributed to prehabilitation, a recent randomized control trial (RCT) showed a 51% reduction of postoperative complications after major abdominal surgery.^[15]^ Given the high morbidity rate, poor initial physical and nutritional status and the need for preoperative chemotherapy, GC patients would be ideal candidates to receive prehabilitation. To date, several studies already investigated the role of prehabilitation in esophagogastric surgery.^[16]^ However, most of these focused on patients receiving esophagectomy, with limited data for gastrectomy.^[16]^ A match pair analysis study showed reduced postoperative morbidity following prehabilitation in patients with GC and metabolic syndrome,^[17]^ while a small pilot study found increased physical status in elderly sarcopenic GC patients.^[18]^ However, these studies were rather small and inconclusive. There is a need for an RCT to address the role of prehabilitation in GC surgery.

The aim of this study is to demonstrate reduced postoperative morbidity after gastrectomy for GC in patients who undergo home-based prehabilitation.

## 2 Methods

### 2.1 Study setting and trial design

**Figure 1:**
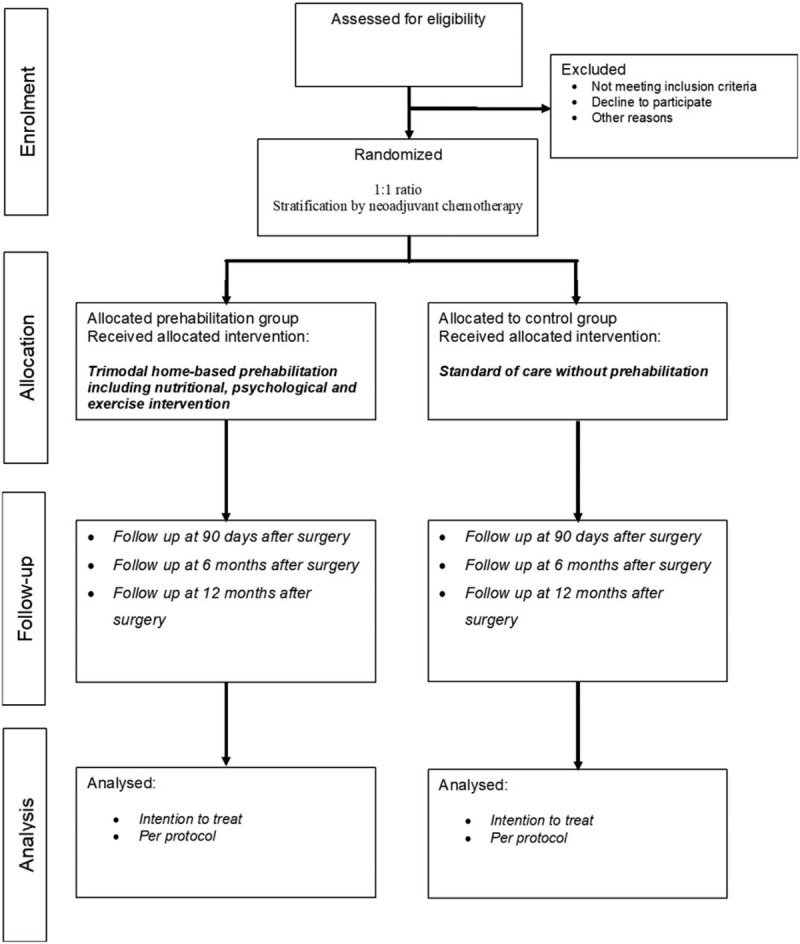
Consort diagram: flow chart.

This multicenter study is designed as a prospective, parallel-group, 1:1 randomized control, open-label trial. The study will be conducted at two major gastrointestinal cancer treatment centers of Lithuania: National Cancer Institute and Vilnius University hospital Santaros Klinikos. A study flowchart is provided in Figure 1. Data collection and follow-up schedules are shown in Table 1.

**Table 1:**
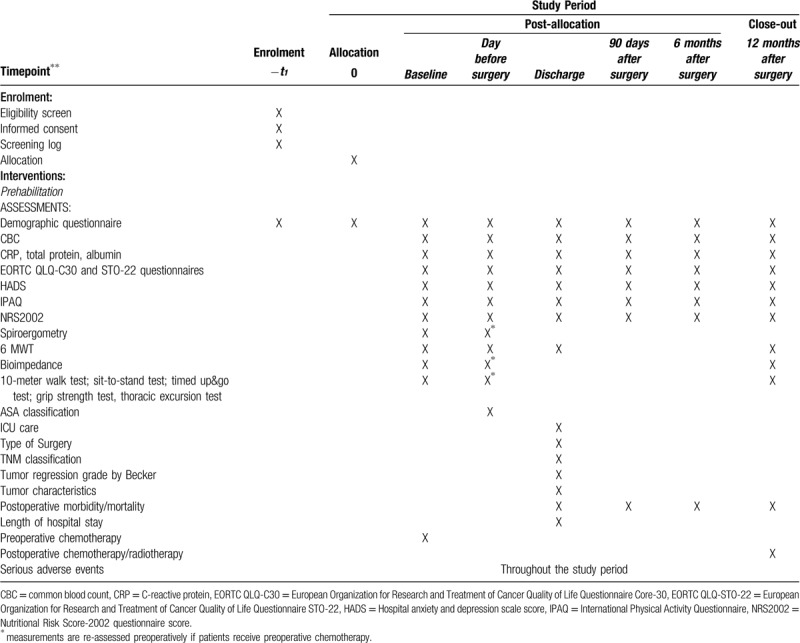
Standard protocol items: recommendations for interventional trials (SPIRIT) figure. Flowchart of the PREhabilitation FOr Gastrectomy (PREFOG) trial.

### 2.2 Eligibility criteria

The study will include gastric cancer patients scheduled for elective total or subtotal gastrectomy at multidisciplinary team meetings. Patients scheduled for gastrectomy first and gastrectomy after neoadjuvant chemotherapy are eligible. Participants will be included after screening for eligibility and obtaining written informed consent. To participate in the study candidates must meet the following inclusion criteria:

1. Age ≥ 18 years.
2. Patient agrees to participate in a clinical study.
3. Patient requires surgical treatment for gastric cancer.

Patients will be excluded if they meet the following criteria:

1. Patient requires surgical treatment for gastric cancer recurrence.
2. Patient condition not allowing to postpone surgery for at least 4 weeks.
3. Patients’ physical or mental condition that does not allow the patient to participate in the prehabilitation program.

### 2.3 Interventions

#### 2.3.1 Control group

The control (standard of care) group will receive routine care from their gastric cancer diagnosis to surgical treatment. These patients will receive no specific advice for prehabilitation before surgery except a recommendation for nutritional supplements by high-energy drinks on the decision of the surgeon.

#### 2.3.2 Prehabilitation group

The prehabilitation (intervention) group will receive home-based trimodal prehabilitation before the gastrectomy. The prehabilitation will consist of:

1) *Exercise intervention:* Patients will be consulted by physical medicine and rehabilitation physicians and physiotherapists to develop personalized home-based exercise program according to a physical performance examination and results of spiroergometry. All patients will undergo three supervised training sessions where they will be trained in correct exercise techniques and self-control in training intensity. Additionally, each patient will receive a written exercise program with detailed description of it. The home-based program will consist of 4 types of exercises:

- Endurance training for 10 to 30 minutes daily by walking/stair climbing/dancing/water exercises/biking. The type of exercise will depend on the patient's choice. The target intensity is 40% to 65% of the heart rate reserve.
- Respiratory muscles training for 5 to 10 minutes daily.
- Resistance training to improve muscular strength for 10 to 20 minutes 3 times per week.
- Stretching exercises for 5 to 10 minutes 3 times per week.

In total daily training sessions will not exceed 60 minutes.

2) *Nutritional support:* A dietician will perform a physical examination and bioimpedance to evaluate the nutritional status of each patient and will provide personalized recommendations for the prevention or correction of malnutrition. The energy and protein requirements will be estimated with 25 to 30 kcal/kg and 1.5 g/kg of ideal body weight respectively. If necessary, patients will be prescribed to consume oral nutritional supplements to increase the consumption of calories and proteins.

3) *Psychological support:* Patients will undergo consultation by specialized onco-psychologist. The anxiety and depression level will be evaluated by the HAD score and patients will be trained to perform relaxation techniques to reduce and manage anxiety at home.

The total length of the prehabilitation program will depend on the gastric cancer treatment pathway. Patients scheduled for the gastrectomy first will undergo prehabilitation for 4 weeks before surgery. Patients scheduled for neoadjuvant chemotherapy first will receive prehabilitation through the entire time of neoadjuvant treatment. The length of the prehabilitation will depend on the neoadjuvant chemotherapy scheme, which is chosen by medical oncologists irrespective of participation in the study.

### 2.4 Strategies to improve adherence to interventions

To increase compliance with the prehabilitation program patients will be asked to fill a diary to record their daily prehabilitation practice. Also, the study staff will contact the patient to inquire about the adherence to the study protocol weekly by the phone call.

### 2.5 Study outcomes

#### 2.5.1 Primary endpoint

The primary outcome of the study is the postoperative morbidity rate by the Clavien-Dindo classification at 90 days postoperatively. All postoperative complications will be recorded at the time of discharge and the surgeon will document any events after discharge at the outpatient appointment 90 days postoperatively. All complications will be classified by the Clavien-Dindo classification.

#### 2.5.2 Secondary endpoints

The secondary endpoints include:

1) Postoperative intrahospital and 90-day mortality rate
2) Postoperative intrahospital and 30-day morbidity rate
3) Physical status of the patient at baseline, pre-surgery, at discharge and 12 months after surgery by:
  - Six-minute walk test
  - Spiroergometry (VO_2,_ VO_2max_, AT)
  - Grip strength test
  - Sit to stand test
  - Timed up and go test
  - Thoracic excursion test
  - International Physical Activity Questionnaire (IPAQ) score.
4) Nutritional status at baseline, pre-surgery, 3, 6 and 12 months after surgery by:
  - Blood albumin level
  - Bioimpedance
  - Nutritional Risk Score-2002 questionnaire score (NRS2002)
5) Quality of Life at baseline, pre-surgery, 3, 6, and 12 months after surgery by:
  - EORTC QLQ-C30 and STO-22 questionnaires scores
6) Anxiety and depression level at baseline, pre-surgery, 3, 6 and 12 months after surgery by:
  - Hospital anxiety and depression scale (HADS) score
7) The proportion of patients completing the oncological treatment fixed in a multidisciplinary tumor board (including preoperative chemotherapy, surgery, and postoperative chemotherapy) at 12 months after treatment initiation

#### 2.5.3 Other objectives and supplementary data collected

Additionally, we will collect data regarding patient demographics, clinical variables (i.e., age and gender, smoking, alcohol usage), surgical and anesthetic details (i.e., intensive care unit and hospital length of stay, type and length of surgery, blood loss, intraoperative complications, t. protein and CRP levels) and disease-related parameters (i.e., stage of disease by TNM classification, tumor regression grade by Becker classification). Study data will be collected and managed using case report forms (CRF).

### 2.6 Biobanking

Additional informed consent may be taken from patients for the biobanking of blood, urine and tissue samples as a part of daily practice in the study institutions. These samples may be used for future laboratory, genetic or molecular analysis.

### 2.7 Data collection and management

All the data will be recorded in CRFs, to maintain the confidentiality all personal data will be coded. Data will be collected at baseline, preoperatively, during the intrahospital period (preoperatively and postoperatively) and after discharge (up until 12 months after surgery).

### 2.8 Recruitment

All gastric cancer patients discussed at the multidisciplinary tumor board meetings in the participating institutions are screened for eligibility for the study. Potentially eligible patients are approached with written informed consent at the outpatient visit to the surgeon. Patient recruitment started at February 2020. Twenty-two months are planned for the recruitment and 300 patients are anticipated to undergo gastrectomy at the study institutions within this time. Therefore, the recruitment of 128 patients seems to be feasible.

### 2.9 Assignment of interventions: allocation

#### 2.9.1 Sequence generation

Participants will be randomly assigned to either control or experimental group with a 1:1 allocation as per a computer-generated randomization schedule stratified by neoadjuvant treatment using random permuted blocks of 4 and 6. The randomization sequence was created using an online available free tool (https://www.sealedenvelope.com/).

#### 2.9.2 Concealment mechanism and implementation

The researcher assistant will prepare sequentially numbered, opaque, sealed envelopes containing randomization sheets. To distinguish patients receiving perioperative chemotherapy, the envelopes will be additionally marked. The randomization sheet will report the randomization code and assigned treatment (standard of care or prehabilitation). The prepared and sealed envelopes will be split into equal piles and delivered to the dedicated place in both study centers. At the time of randomization, the investigator (surgeon consulting the patient) will choose an envelope with the lowest number and will write the name and date of birth of the participant before opening to prevent subversion of the allocation sequence. After opening, the randomization information will be given to the patient, the baseline condition of the patient will be assessed and the case report form (CRF) will be filled in.

#### 2.9.3 Blinding

The study cannot be blinded because participation in a prehabilitation program cannot be hidden from neither patients nor practitioners.

### 2.10 Sample size

The sample size calculation was done using G∗Power 3.1.9.4 software using the reduction of 90 days postoperative complication rates as the primary outcome. Based on the assumption that the percentage of patients developing postoperative complications after gastrectomy is approximately 50% for the control group (based on our centers historical experience and results from RCTs)^[2,3]^ and can be reduced to 25% in the prehabilitation group (based on results of recent RCT showing 50% reduction of postoperative complications by prehabilitation),^[15]^ a group sample size of 58 patients is needed to achieve 80% power in detecting this difference in 90-days postoperative morbidity at a two-sided level of significance of 5%. Under the assumption of a drop-out rate of up to 10%, a total of 128 patients (64 per group) needs to be enrolled in the study.

### 2.11 Statistical analysis

All clinical data will be analyzed on an intention to treat basis but will also be described on ‘as treated’ basis. Initially, all the clinical data will be analyzed using descriptive statistic methods. The primary outcome analysis will be based on a Chi-Square test. For secondary endpoint analysis, Chi-Square or Fisher Exact test, T-Test or Mann-Whitney test will be used where appropriate. Other statistical methods will be used if there will be a need.

### 2.12 Criteria for discontinuing or modifying allocated intervention

The study can be terminated for individual patients due to:

(a) a severe adverse event;
(b) significant protocol violations;
(c) withdrawal of consent;
(d) loss to follow-up;
(e) any other situation that leads to the decision to terminate the study.

The whole trial can be stopped by the investigator if adverse events occur or other unforeseeable events might influence the safety or well-being of the study participants. After termination, all study patients will be followed up according to the standard follow-up policy of our institution for gastric cancer.

### 2.13 Ethics

The study protocol has been approved by the Vilnius University Regional Bioethics committee (Nr.2020/1-1185-675) and registered with clinicaltrials.gov (NCT04223401). Written informed consent will be obtained from the patients before participation in the study. The trial will be performed guided by the World Medical Association's Declaration of Helsinki, Guideline for Good Clinical Practice, and regulatory laws in Lithuania.

## 3 Discussion

Surgical resection remains the cornerstone of treatment with curative intent for GC. However, postoperative complications after gastrectomy are a significant problem resulting in increased treatment costs, prolonged hospital stay, delayed adjuvant therapy and impaired long-term outcomes.^[19–22]^ The physical, nutritional and emotional capacity of the individual patients predicts the postoperative outcomes.^[23–26]^ Some of the interventions, such as intensive intraoperative monitoring, well-timed admission to intensive care unit and enhanced recovery through ERAS protocols are proposed in the early perioperative period to improve the postoperative outcomes.^[27]^ Although, the ideal timeframe for prehabilitation intervention is the preoperative period, as the decline in physical and emotional status is to be expected after major surgery. An intensive postoperative program would be more detrimental to patient recovery and would fail to better prepare patients for surgery. It is rational to expect, that increasing patients’ physiological fitness before surgical trauma will preserve a higher level of functional capacity over the entire perioperative period and would hopefully extend postoperatively. The process of improving patients’ physical, nutritional, and emotional capacity before surgery has been termed multimodal prehabilitation. The goal centers on wholesome preparation of the patient to withstand the physical and emotional stress of surgery.^[28]^*Promising results of prehabilitation have been reported including reduced* postoperative complication rates^[15]^ and earlier recovery of physical function after major abdominal surgery.^[29]^ Although the current evidence from randomized studies remains weak and inconsistent, it suggests a potential strategy to reduce postoperative mortality rates.^[30,31]^ Only a few studies focused on prehabilitation for gastrectomy and none of them investigated the real multimodal prehabilitation approach in a randomized control trial.^[16,17]^ The matched-pair analysis by Cho et al^[17]^ showed the benefit of isolated exercise intervention for GC patients with a high body mass index (>25 kg/m^2^) and metabolic syndrome, while the pilot study by Yamamoto et al^[18]^ demonstrated the potency of exercise and nutritional intervention for sarcopenic and elderly GC patients. Despite this promising potential to improve postoperative outcomes by prehabilitation, the evidence level is low and further investigation is necessary. Currently, multimodal prehabilitation including exercise, nutritional and psychological interventions is under investigation in the ongoing PREHAB study.^[32]^ However, this study employs supervised exercise interventions, which require repeated clinical appointments. It becomes a geographical challenge when the cancer centers cover a large area with a widely spread population. The need for routine visits limits patients’ recruitment and adherence. To overcome such logistic issues, home-based prehabilitation was considered as an alternative, especially as recent data support the effectiveness of such programs for patients with lung and pancreatic cancer.^[33,34]^ Thus, our study was designed to investigate the trimodal prehabilitation in a home-based setting to limit participant visits, improve recruitment and reduce participant burden. Naturally, the compliance to the prehabilitation program in a home-based setting may be challenging. Therefore, the first three exercise trainings are supervised by a physiotherapist to ensure appropriate techniques and provide detailed written instructions for further personalized training at home. To assure compliance to the program we will use a self-reported diary and will implement a weekly phone call to assess patient adherence. The PREFOG study presented here will demonstrate whether home-based personalized prehabilitation will decrease the postoperative morbidity after gastrectomy for gastric cancer. Moreover, this study will allow us to determine whether prehabilitation can improve physical fitness and activity levels, nutritional status and quality of life, as well as reduce anxiety and depression levels after gastrectomy for gastric cancer.

## 4 Declarations

We declare that this study is funded by Vilnius University. The individual data of the patients will remain confidential. The results of this study may be presented at national and international conferences and published. The study is a part of doctoral thesis of A.B. at the Faculty of Medicine Vilnius University. All the authors declare that they have no additional conflict of interest.

Protocol date and version and study status: Protocol version 2, dated 2020-01-29. Currently study is recruiting patients.

## Author contributions

All listed authors contributed contributed to the conception and design of the work. KS, RB, SL, EG JC contributed to the provision of resources; AB, ML, JK, GA, VM, LG, IJ, AC, DV and GK contributes to the acquisition of data. AB prepared the manuscript. RB and KS revised the manuscript. All authors read and approved the final manuscript.
